# P75 *Vibrio cholerae* bacteraemia in an immunocompetent returning traveller

**DOI:** 10.1093/jacamr/dlag102.081

**Published:** 2026-06-26

**Authors:** Sachika Paththamperuma, Linda Loterh, Madhuri Vidwans

**Affiliations:** Department of Microbiology, Watford General Hospital, Watford, UK; Department of Microbiology, Watford General Hospital, Watford, UK; Department of Microbiology, Watford General Hospital, Watford, UK

## Abstract

**Background:**

*Vibrio cholerae* is a Gram-negative, comma-shaped bacterium that causes cholera, an acute diarrhoeal illness that remains endemic in many parts of Africa and South Asia. While it is well recognized as a cause of severe diarrhoeal disease, *V. cholerae* bacteraemia is rare, particularly in immunocompetent individuals. We present a case of *V. cholerae* bacteraemia in an immunocompetent returning traveller.

**Case Presentation:**

A 54-year-old female with a background history of hypertension presented with a 3 day history of profuse watery diarrhoea, vomiting, and fever. She had returned from Tanzania 10 days prior to admission, where she had stayed in hotels and consumed street food. Her husband also reported mild gastrointestinal symptoms following the trip. On admission, the patient was febrile and clinically dehydrated. She was haemodynamically stable, with a normal blood pressure and a heart rate of 88 beats per minute. Initial laboratory investigations revealed a white blood cell count of 5.62 ×10⁹/L, with a neutrophil count of 2.40 ×10⁹/L, and a markedly elevated C-reactive protein (CRP) of 109 mg/L. Biochemical investigations demonstrated electrolyte disturbances, including hypokalaemia, hypocalcaemia, and hypomagnesaemia, along with mildly deranged liver function tests. Blood cultures obtained on admission subsequently flagged positive in both bottles, showing Gram-negative bacilli on microscopy. The organism was identified as *V. cholerae* using MALDI-TOF mass spectrometry, with confirmation by the reference laboratory. Stool culture obtained at admission was negative. The patient was isolated in a side room, and standard as well as enteric infection prevention precautions were implemented. Empirical antimicrobial therapy was initially escalated to IV ceftriaxone. Following microbiological confirmation, treatment was rationalized to azithromycin, which was administered for 3 days. The patient demonstrated significant clinical improvement with antimicrobial therapy and supportive management, including fluid and electrolyte replacement, and was subsequently discharged home.

**Discussion:**

*V. cholerae* infection is primarily associated with gastrointestinal disease; however, invasive infections such as bacteraemia are uncommon and typically reported in immunocompromised individuals or those with underlying co morbidities such as liver disease. This case highlights the unusual occurrence of *V. cholerae* bacteraemia in an immunocompetent host. Early recognition of *V. cholerae* infection is crucial, particularly in returning travellers from endemic regions such as Sub-Saharan Africa. A detailed travel history is essential in guiding appropriate microbiological investigations and clinical management. Interestingly, stool cultures in this case were negative despite confirmed bacteraemia, emphasizing the importance of obtaining blood cultures in patients presenting with systemic features of infection. Rapid identification methods such as MALDI-TOF mass spectrometry play a key role in timely diagnosis and targeted antimicrobial therapy. Appropriate infection control measures, prompt antimicrobial treatment, and aggressive supportive care, particularly fluid and electrolyte replacement, are essential in ensuring favourable clinical outcomes.

**Conclusions:**

This case represents a rare occurrence of *V. cholerae* bacteraemia in an immunocompetent individual. Early microbiological diagnosis, combined with prompt and appropriate antimicrobial therapy and supportive management, is crucial in preventing complications and ensuring favourable patient outcomes.

